# Structural Insights Into centSIRT6: Bioinformatic Analysis of N308K and A313S Substitution Effects

**DOI:** 10.1177/11779322251339698

**Published:** 2025-05-21

**Authors:** Francisco Alejandro Lagunas-Rangel

**Affiliations:** 1 Department of Surgical Sciences, Uppsala University, Uppsala, Sweden; 2 Laboratory of Pharmaceutical Pharmacology, Latvian Institute of Organic Synthesis, Riga, Latvia

**Keywords:** Sirtuin, docking, acetyl-lysine, active site, substrate-enzyme complex energy

## Abstract

Sirtuin 6 (SIRT6), a member of the class III histone deacetylase (HDAC) family, is crucial for the maintenance of general health and is associated with increased life expectancy and resistance to age-related diseases such as cancer and metabolic disorders. A comparative analysis of the SIRT6 gene in Ashkenazi Jewish (AJ) centenarians and noncentenarian controls found a distinct allele, centSIRT6, enriched in the centenarian group. This allele features 2 linked substitutions, N308K and A313S, and exhibits enhanced functions, including more efficient suppression of LINE1 retrotransposons, improved repair of DNA double-strand breaks, and increased efficiency in cancer cell killing. Notably, centSIRT6 shows lower deacetylase activity but higher mono-adenosine diphosphate (ADP) ribosyl transferase activity compared with the wild-type enzyme. This study used several bioinformatics tools to explore the structural changes caused by the N308K and A313S substitutions in centSIRT6 and to elucidate how these alterations contribute to changes in the enzymatic activities of SIRT6. The results indicate that these mutations reduce the structural flexibility of centSIRT6, thus weakening its interactions with acetyl-lysine but strengthening its interactions with ADP-ribose. This research provides useful information for future experimental studies to further investigate the molecular mechanisms of centSIRT6.

## Introduction

Sirtuin 6 (SIRT6) is a member of the class III histone deacetylase (HDAC) family, commonly known as sirtuins.^1^ Phylogenetic studies further classify SIRT6, along with SIRT7, into the class IV subgroup of this family.^2,3^ It is primarily localized in the nucleus, highlighting their critical roles in regulating processes such as DNA repair, transcription, and genome stability.^4^ SIRT6 has attracted significant attention for its vital contribution to maintaining physiological health. It has been implicated in lifespan extension and exhibits protective effects against aging, cancer, and metabolic disorders. Elevated SIRT6 levels have been associated with enhanced metabolic function, increased longevity, and resistance to age-related diseases.^5^

Although SIRT6 is primarily recognized for its deacetylase activity, this function is relatively weak compared with other sirtuins.^6^ Its main biological roles are largely attributed to its adenosine diphosphate (ADP)-ribosylation activity, particularly in pathways related to DNA repair and genome stability.^7^ These enzymatic reactions, which target lysine residues, require oxidized nicotinamide adenine dinucleotide (NAD⁺) as a co-substrate. Key substrates of SIRT6 include acetylated lysines on histone H3, notably H3K9ac, H3K56ac, and H3K18ac.^8-10^ The removal of these chromatin marks by SIRT6 is crucial for promoting chromatin compaction, repressing transcription, and initiating DNA damage response pathways.^11^

Beyond histone targets, SIRT6 also modifies several nonhistone proteins. It deacetylates factors involved in DNA repair and glucose metabolism,^12^ catalyzes mono-ADP-ribosylation of DNA repair proteins^13^ and chromatin silencing,^14^ and removes long-chain fatty acyl groups from substrates such as tumor necrosis factor α (TNFα).^15^ These diverse enzymatic functions highlight the central role of SIRT6 in preserving genomic integrity, regulating metabolic processes, and promoting cellular health.

A comparative study analyzing the SIRT6 gene in Ashkenazi Jewish (AJ) centenarians and AJ controls with no family history of exceptional longevity identified a specific SIRT6 allele enriched in the centenarian group.^16^ This allele, denoted centSIRT6, has 2 linked substitutions (N308K and A313S) and was shown to provide benefits compared with wild-type SIRT6. The centSIRT6 demonstrated superior suppression of long interspersed nuclear element-1 (LINE1) retrotransposons, enhanced stimulation of DNA double-strand break repair, and a more robust ability to kill cancer cells.^16-18^ Interestingly, centSIRT6 showed weaker deacetylase activity than its wild-type counterpart but higher mono-ADP ribosyl transferase activity.^16^

With this context, the aim of the study was to employ bioinformatics tools to investigate the structural changes caused by the N308K and A313S substitutions in centSIRT6, in comparison to the wild-type SIRT6. By analyzing these alterations, the study sought to shed light on how these mutations contribute to the reduced deacetylase activity and the enhanced mono-ADP-ribosyltransferase activity observed in the centSIRT6 variant.

## Materials and Methods

### Sequence analysis and substitution impact assessment

The wild-type SIRT6 sequence was retrieved from the UniProt Knowledgebase (UniProtKB).^19^ The centSIRT6 sequence was generated by manually modifying the wild-type sequence as needed to introduce the desired changes (N308K and A313S). To predict the potential effects of amino acid changes on SIRT6 function, the sorting intolerant from tolerant (SIFT) tool^20^ was used, using its default parameters. In addition, to assess the impact of substitutions on protein conformation, flexibility, and stability, DynaMut^21^ was applied, also using its default parameters. I-Mutant2.0^22^ was also employed using its default parameters.

### Protein modeling, validation, and docking

**Figure 1.**
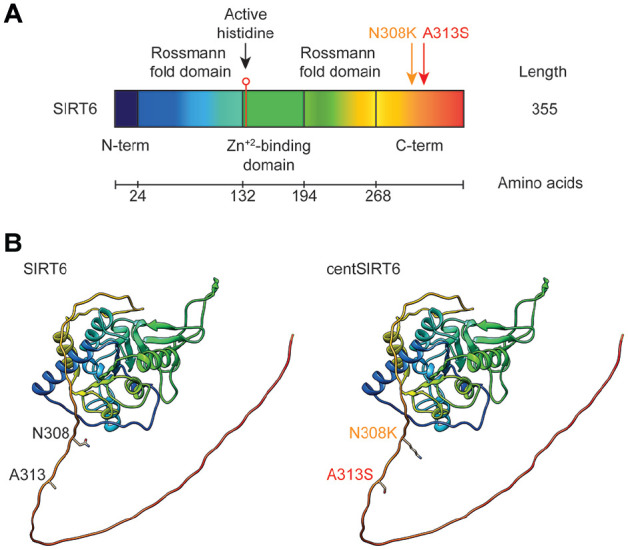
Comparative schematic of wild-type SIRT6 and centSIRT6 variants. (A) Diagram showing the domain organization of wild-type SIRT6. (B) Three-dimensional structural models of the centSIRT6 and wild-type SIRT6 proteins, highlighting the positions of the N308K and A313S mutations.

Three-dimensional (3D) models of wild-type SIRT6 and centSIRT6 (with both substitutions: N308K and A313S) were predicted using AlphaFold,^23^ and visualizations were generated with UCSF Chimera software^24^ (detailed sequences can be found in Supplementary File 1). Figure 1 provides a schematic overview of the SIRT6 domains, highlighting the mutation sites in centSIRT6 and displaying the 3D structural models of both SIRT6 and centSIRT6. The quality of the generated 3D structures was evaluated with PROCHECK,^25^ specifically by examining the Psi/Phi angles in the Ramachandran diagram (Supplementary File 1). Docking simulations were performed with SwissDock, using the attracting cavities 2.0 method.^26^ Acetyl-lysine (ZINC3200634) and ADP-ribose (ZINC32786513), obtained from the ZINC20 database,^27^ were used as ligands in these studies.

### Molecular dynamics simulation

Molecular dynamics (MD) simulations were conducted following the approach outlined in previous studies,^3,28,29^ utilizing the AMBER20 software package for the simulations.^30^ Hydrogen atoms were added to the protein structures using the *tleap* module, applying the ff14SB force field.^31^ Force field parameters for all compounds were generated with the Antechamber module using the AM1-BCC charge model.^32^ Each system was solvated in a TIP3P water box,^33^ and neutralized by adding chloride ions. Energy minimization was carried out in 2 stages: 4000 steps using the steepest descent method, followed by 1000 steps of conjugate gradient minimization. The systems were gradually heated from 0 to 300 K under constant pressure (NPT ensemble) over 30 ps, applying weak positional restraints (force constant k = 10 kcal/mol·Å^2^) to the proteins. Subsequently, 100 ns of unrestrained MD simulations were performed at 300 K in the NVT ensemble, using a 2-fs time step. Temperature control was maintained using a Langevin thermostat, and pressure was regulated with the anisotropic Berendsen barostat. Structural stability and flexibility were assessed by calculating root-mean-square deviations (RMSDs) and root-mean-square fluctuations (RMSF) relative to the initial conformations.

## Results

### N308K and A313S substitutions reduce SIRT6 flexibility

In order to assess the potential effects of the N308K and A313S substitutions in centSIRT6 and to determine whether they could alter its enzymatic function, an analysis was performed using SIFT. This tool evaluates sequence homology and physical properties of amino acids to predict functional impacts. The analysis revealed that the N308K substitution is predicted to significantly affect protein function, with a score of <0.010, indicating a deleterious effect. In contrast, the A313S substitution is predicted to be tolerated, with a score of 0.050.

To further analyze the effects of substitutions, we used DynaMut, a computational tool that combines graph-based signatures with normal mode dynamics to predict the impact of substitutions on protein stability. The overall change in Gibbs free energy (ΔΔG) for the N308K substitution was 0.161 kcal/mol, indicating a slight stabilizing effect. Detailed predictions included elastic network contact model (ΔΔG ENCoM) of 0.070 kcal/mol, mutation cutoff scanning matrix (ΔΔG mCSM) of −0.007 kcal/mol, site-directed mutator (ΔΔG SDM) of 0.180 kcal/mol, and a consensus predictor combining mCSM and SDM (ΔΔG DUET) of 0.398 kcal/mol. Vibrational entropy energy analysis revealed a ΔΔS_Vib_ ENCoM of −0.088 kcal/(mol*K), suggesting a minor reduction in protein flexibility. These results suggest that the N308K substitution has a slight stabilizing effect on the protein while marginally decreasing its flexibility.

For the A313S substitution, the overall ΔΔG was −0.703 kcal/mol, indicating a destabilizing effect. In detail, ΔΔG ENCoM was 0.117 kcal/mol, ΔΔG mCSM −0.668 kcal/mol, ΔΔG SDM −0.900 kcal/mol, and ΔΔG DUET −0.493 kcal/mol. Furthermore, ΔΔS_Vib_ ENCoM prediction for this substitution was −0.146 kcal/(mol*K), suggesting a decrease in molecular flexibility. These results indicate that the A313S substitution significantly destabilizes the protein and reduces its flexibility.

To support the DynaMut findings, I-Mutant2.0, an support vector machine (SVM)-based tool for predicting protein stability changes upon single-point mutations was also used. The tool predicted a slight decrease in stability for both mutations, with a reliability index (RI) of 1.000 for N308K and 7.000 for A313S.

### N308K and A313S substitutions weaken the interactions between centSIRT6 and acetyl-lysine

**Figure 2.**
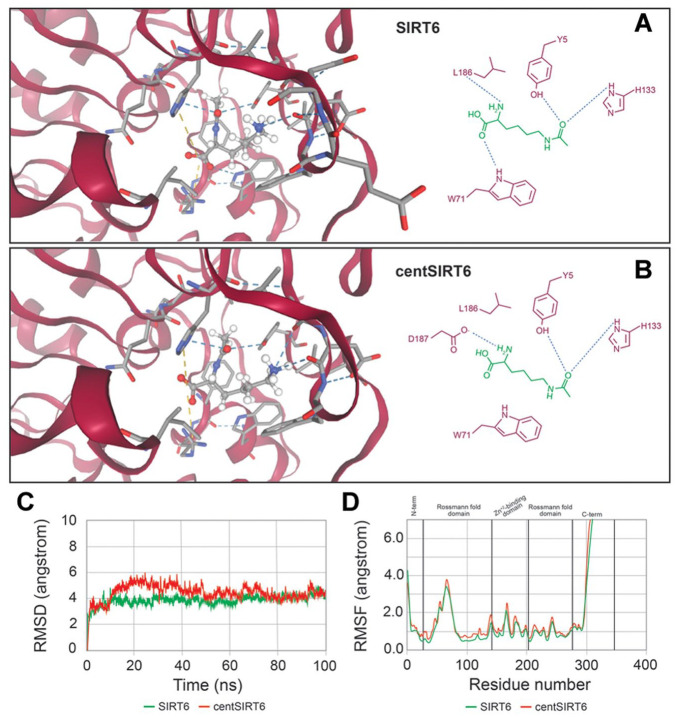
SIRT6 and centSIRT6 with their substrate acetyl-lysine. Panel (A) shows the interaction of acetyl-lysine with SIRT6, whereas panel (B) depicts the complex with centSIRT6. Key amino acids involved in substrate binding are highlighted. Panel (C) presents a comparative analysis of the root-mean-square deviations (RMSD) from molecular dynamics simulations of wild-type SIRT6 and centSIRT6 with acetyl-lysine, while (D) displays the corresponding root-mean-square fluctuations (RMSFs).

To investigate the structural changes caused by the N308K and A313S substitutions, a docking study was performed. The results revealed altered interactions between centSIRT6 and its substrate, acetyl-lysine, relative to the wild-type protein (Figure 2A and B). The total energy of the substrate-enzyme complex in centSIRT6 was less negative (−49.215 kcal/mol) compared with the wild-type complex (−55.593 kcal/mol). This indicates that the complex with the mutated protein is less stable than with the original protein. Similarly, the free-binding energy in centSIRT6 was less favorable (−5.793 kcal/mol) compared with the wild type (−6.205 kcal/mol). This change reflects that substitutions have reduced the spontaneity of the interaction.

A detailed analysis showed remarkable differences in the interaction contributions: the polar contribution in centSIRT6 was higher (14.451 kcal/mol) than in the wild type (12.023 kcal/mol), whereas the nonpolar contributions were less favorable (−28.411 kcal/mol in centSIRT6 vs −31.192 kcal/mol in the wild type). The polar contribution implies that polar interactions are less favorable and raises the energy of the system. Conversely, the contribution of nonpolar interactions reflects a reduction of hydrophobic and Van der Waals interactions, which may explain the lower affinity of the ligand. The total interaction energy due to substitutions was less negative in centSIRT6 (−13.960 kcal/mol) compared with the wild type (−19.000 kcal/mol). This means weaker overall interactions. In addition, the polar15 correction was less favorable in centSIRT6 (−6.734 kcal/mol) than in the wild type (−13.158 kcal/mol). The RMSD of the substrate conformation was slightly reduced in centSIRT6 compared with the wild type. However, MD simulations revealed that wild-type SIRT6 maintains a more stable and consistent conformation throughout the simulation compared with centSIRT6 (Figure 2C and D).

### N308K and A313S substitutions enhance centSIRT6-ADP-ribose interactions

**Figure 3.**
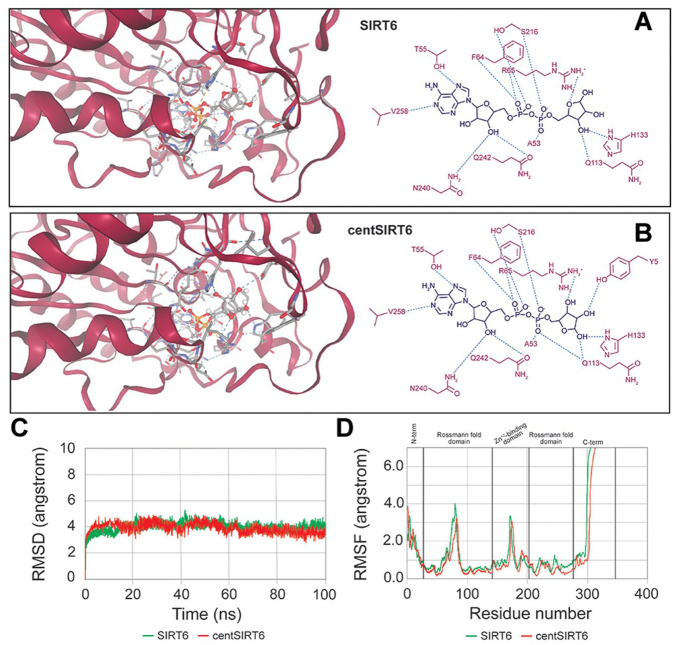
SIRT6 and centSIRT6 with their substrate ADP-ribose. Panel (A) shows the interaction of ADP-ribose with SIRT6, whereas panel (B) depicts the complex with centSIRT6. Key amino acids involved in substrate binding are highlighted. Panel (C) presents a A comparative analysis of the root-mean-square deviations (RMSD) from molecular dynamics simulations of wild-type SIRT6 and centSIRT6 with ADP-ribose, while (D) displays the corresponding root-mean-square fluctuations (RMSFs).

The centSIRT6 adopts a conformation similar to wild-type SIRT6 when bound to ADP-ribose, but this conformation is more likely and more stable (Figure 3A and B). The total energy of the substrate-enzyme complex in centSIRT6 is more negative (−178.830 kcal/mol) compared with the wild-type complex (−113.012 kcal/mol). This indicates that the complex with the protein with substitutions is more stable than with the original protein. Similarly, the free binding energy in centSIRT6 is more favorable (−11.186 kcal/mol) than in the wild type (−11.183 kcal/mol). A more detailed analysis revealed differences in interaction contributions between centSIRT6 and the wild-type structure. In centSIRT6, the polar contribution was lower, 12.919 kcal/mol, compared with 14.644 kcal/mol in the wild type. It decreases system energy. Meanwhile, the nonpolar contributions were less favorable, measuring −84.428 kcal/mol in centSIRT6 vs −85.513 kcal/mol in the wild type and implying an increase in hydrophobic and Van der Waals interactions. The total interaction energy associated with substitutions was more negative in centSIRT6 structure, calculated at −71.508 kcal/mol, compared with −70.868 kcal/mol in the wild type. Similarly, the polar correction term was more favorable in centSIRT6 (−71.508 kcal/mol) than in the wild type (−70.868 kcal/mol). The RMSD of the substrate conformation is slightly increased in centSIRT6 compared with the wild type. The MD simulations showed that while these minimal changes initially occur, they are eventually reversed, with centSIRT6 maintaining a more stable and consistent conformation throughout the simulation compared with wild-type SIRT6 (Figure 3C and D).

## Discussion

The catalytic core of human SIRT6 spans residues 27-272 and, like other sirtuins, has an elongated structure comprising a large folded Rossmann domain, typical of NAD⁺/NADH-binding proteins, and a smaller zinc-binding domain. Zinc-binding sites are located at residues C141, C144, C166, and C177, while residue H133 of the catalytic domain is a key proton acceptor in deacetylation activity (Figure 1).^34^ Notably, SIRT6 and SIRT7, unlike other sirtuins, lack a large helical bundle within the NAD+-binding Rossmann fold that typically links the catalytic and zinc-binding domains. This absence is thought to reduce structural flexibility, contributing to their inherently low deacetylase activity.^35,36^

N308K and A313S substitutions in centSIRT6 are predicted to result in reduced structural flexibility compared with wild-type SIRT6. These structural changes decrease the ability of the enzyme to interact with acetyl-lysine, mainly due to a reduction in total binding energy and a decrease in binding affinity. Conversely, these alterations strengthen the enzyme’s interactions with ADP-ribose, probably by stabilizing the binding pocket and improving the enzyme’s compatibility with this substrate. Consequently, it is possible that these factors combined drive the observed reduction in centSIRT6 deacetylase activity while enhancing its mono-ADP ribosyl transferase activity. Table 1 provides a summary of the results from the various tools used in this study. Notably, none of the substitutions are located near the catalytic site but rather at the C-terminal end of SIRT6. This C-terminal region is highly flexible, and its structure has not been experimentally resolved due to its dynamic nature.^34^ Since the C-terminal region of SIRT6 plays a role in mediating interactions with other proteins, substitutions not only alter the catalytic site but may also affect protein-protein interactions. These modifications could affect the broader functional network of SIRT6 and its interaction dynamics with its cellular partners.^37^ A recent preprint, based on MD simulations, suggests that the C-terminal region of SIRT6 adopts multiple conformations, some of which facilitate DNA unwrapping. This structural flexibility enhances the accessibility of H3K27 to the enzyme’s active site, supporting a regulatory role for the C-terminal domain in substrate recognition and deacetylation.^38^ Clearly, the geometry and flexibility of acetyl-lysine and ADP-ribose also contribute to the observed changes in centSIRT6 enzymatic activities.

**Table 1.** Summary of results obtained using different bioinformatics tools.

Bioinformatic tool	Substitution		Result
SIFT	N308K		Score < 0.010
	A313S		Score = 0.050
DynaMut	N308K		ΔΔG = 0.161 kcal/mol ΔΔG ENCoM = 0.070 kcal/mol ΔΔG mCSM = −0.007 kcal/mol ΔΔG SDM = 0.180 kcal/mol ΔΔG DUET = 0.398 kcal/mol ΔΔS_Vib_ ENCoM = −0.088 kcal/(mol*K)
	A313S		ΔΔG = −0.703 kcal/mol ΔΔG ENCoM = 0.117 kcal/mol ΔΔG mCSM = −0.668 kcal/mol ΔΔG SDM = −0.900 kcal/mol ΔΔG DUET = −0.493 kcal/mol ΔΔS_Vib_ ENCoM = −0.146 kcal/(mol*K)
I-Mutant2.0	N308K		Stability decreased (RI = 1)
	A313S		Stability decreased (RI = 7)
Bioinformatic tool	Protein	Ligand	Result
Docking (SwissDock)	SIRT6	Acetyl-lysine	MEMBER_ENERGY: −55.593164 SP-dG: −6.2050 Polar: 12.0229 Nonpolar: −31.1921 Inter: −19.1692 Polar15: −13.1578
	centSIRT6		MEMBER_ENERGY: −49.215205 SP-dG: −5.7933 Polar: 14.4508 Nonpolar: −28.4106 Inter: −13.9598 Polar15: −6.7344
	SIRT6	ADP-ribose	MEMBER_ENERGY: −173.012546 SP-dG: −11.1833 Polar: 14.6444 Nonpolar: −85.5133 Inter: −70.8689 Polar15: −63.5467
	centSIRT6		MEMBER_ENERGY: −178.830018 SP-dG: −11.1863 Polar: 12.9199 Nonpolar: −84.4287 Inter: −71.5087 Polar15: −65.0488

These results include changes in Gibbs free energy (ΔΔG) from various prediction methods: Elastic Network Contact Model (ΔΔG_ENCoM), Mutation Cutoff Scanning Matrix (ΔΔG mCSM), Site-Directed Mutator (ΔΔG SDM), and a consensus predictor combining mCSM and SDM (ΔΔG DUET). The vibrational entropy change is represented as ΔΔS_Vib_ ENCoM. Molecular docking and binding energy descriptors include estimated binding energy (MEMBER_ENERGY), fullFitness (SP-dG), polar interactions (Polar), no polar interactions (Nonpolar), total intermolecular interaction energy between the ligand and the target protein (Inter), the polar interaction energy within a 15-Å radius of the binding site (Polar15), and root-mean-square deviation (RMSD).

The centSIRT6 showed a markedly increased ability to suppress LINE-1 retrotransposons, significantly greater stimulation of DNA double-strand break repair, and demonstrated an enhanced ability to induce cancer cell death.^16^ Beyond its cellular effects, centSIRT6 overexpression produces physiological effects such as improved glucose sensitivity and modulates sympathetic innervation signaling in mature adipocytes, contributing to improved metabolic regulation.^17^ In hepatocytes, overexpression of centSIRT6 leads to increased levels of most amino acids, unsaturated fatty acids, and glycerophospholipids while reducing ceramide levels. These changes reflect a shift toward a healthier metabolic profile.^18^

Although predictive models suggest a slight decrease in stability due to the substitutions in centSIRT6, the differences were minimal across all analyses. In addition, SIRT6 is not continuously active; only a portion of the protein is functionally engaged at any given time, with its activity finely regulated by factors such as NAD⁺ availability, which is closely linked to the organism’s nutritional and metabolic state.^39^ As a result, a reserve pool of inactive SIRT6 remains available for activation, and transcriptional compensation may help sustain adequate functional enzyme levels.^40^ What this manuscript mainly highlights is the change in the levels of their enzymatic activities, both deacetylation and ADP-ribosylation.

During aging, histone and nonhistone acetylation levels decrease as a consequence of age-related metabolic changes, including reduced acetyl-CoA and citrate concentrations.^41^ These metabolic changes alter transcriptional regulation and impair the efficiency of DNA repair pathways.^41,42^ In this context, decreasing the acetylase activity of SIRT6 in centSIRT6 could be advantageous, as it could help to restore balance and mitigate the negative effects of reduced acetylation levels. Meanwhile, mono-ADP-ribosylation plays an important role in the regulation of diverse cellular pathways and is closely related to general health and the development of numerous diseases.^43^ The increased mono-ADP ribosyl transferase activity of centSIRT6 could be particularly advantageous in modulating processes such as cell cycle progression, DNA damage repair, inflammatory responses, and defence mechanisms against viral infections and cancer, among others. This functional gain could significantly enhance cellular resilience and may underlie the better health and extended lifespan observed in individuals carrying the centSIRT6 variant.^16^

## Limitations of the Study

This study is based solely on data generated through bioinformatics analyses, which rely on predictive models and computational tools. As a result, the findings presented should be interpreted as preliminary and theoretical in nature. To confirm the accuracy and relevance of these predictions, experimental validation is essential in future research.

## Supplemental Material

sj-docx-1-bbi-10.1177_11779322251339698 – Supplemental material for Structural Insights Into centSIRT6: Bioinformatic Analysis of N308K and A313S Substitution Effects Supplemental material, sj-docx-1-bbi-10.1177_11779322251339698 for Structural Insights Into centSIRT6: Bioinformatic Analysis of N308K and A313S Substitution Effects by Francisco Alejandro Lagunas-Rangel in Bioinformatics and Biology Insights
