# Supplementary material for: Structural Insights Into centSIRT6: Bioinformatic Analysis of N308K and A313S Substitution Effects
Source: Bioinform Biol Insights. 2025 May 21;19:11779322251339698. doi: 10.1177/11779322251339698 (PMC12099093; doi:10.1177/11779322251339698)

**Sequences**

**SIRT6 wild-type**

MSVNYAAGLSPYADKGKCGLPEIFDPPEELERKVWELARLVWQSSSVVFHTGAGISTASGIPDFRG

PHGVWTMEERGLAPKFDTTFESARPTQTHMALVQLERVGLLRFLVSQNVDGLHVRSGFPRDKLAE

LHGNMFVEECAKCKTQYVRDTVVGTMGLKATGRLCTVAKARGLRACRGELRDTILDWEDSLPDRD

LALADEASRNADLSITLGTSLQIRPSGNLPLATKRRGGRLVIVNLQPTKHDRHADLRIHGYVDEVMT

RLMKHLGLEIPAWDGPRVLERALPPLPRPPTPKLEPKEESPTRI**N**GSIP**A**GPKQEPCAQHNGSEPAS

PKRERPTSPAPHRPPKRVKAKAVPS **centSIRT6**

MSVNYAAGLSPYADKGKCGLPEIFDPPEELERKVWELARLVWQSSSVVFHTGAGISTASGIPDFRG

PHGVWTMEERGLAPKFDTTFESARPTQTHMALVQLERVGLLRFLVSQNVDGLHVRSGFPRDKLAE

LHGNMFVEECAKCKTQYVRDTVVGTMGLKATGRLCTVAKARGLRACRGELRDTILDWEDSLPDRD

LALADEASRNADLSITLGTSLQIRPSGNLPLATKRRGGRLVIVNLQPTKHDRHADLRIHGYVDEVMT

RLMKHLGLEIPAWDGPRVLERALPPLPRPPTPKLEPKEESPTRI**K**GSIP**S**GPKQEPCAQHNGSEPAS

PKRERPTSPAPHRPPKRVKAKAVPS


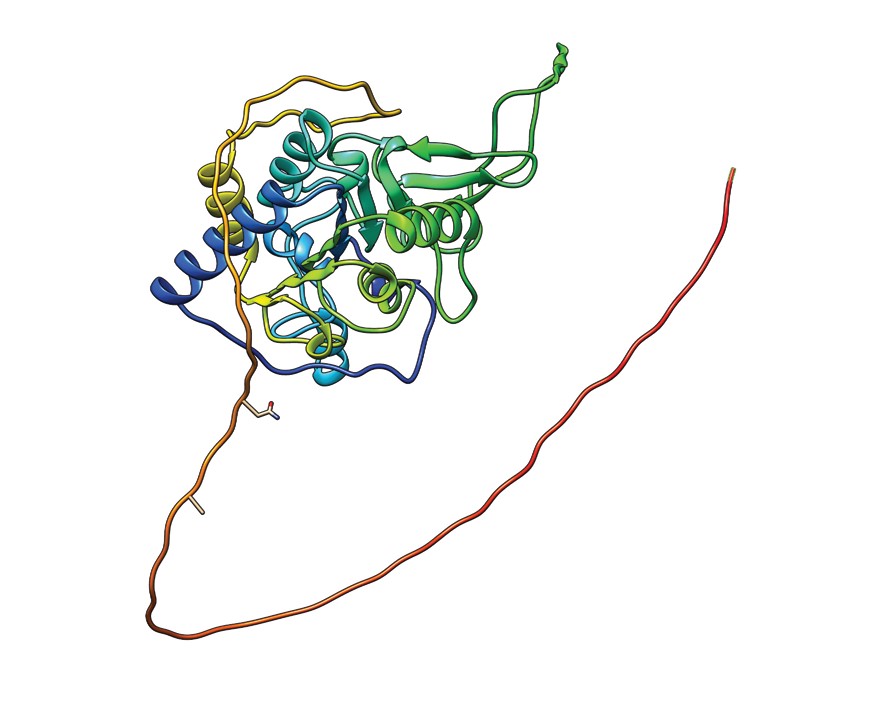


SIRT6 wild-type

N308

A313


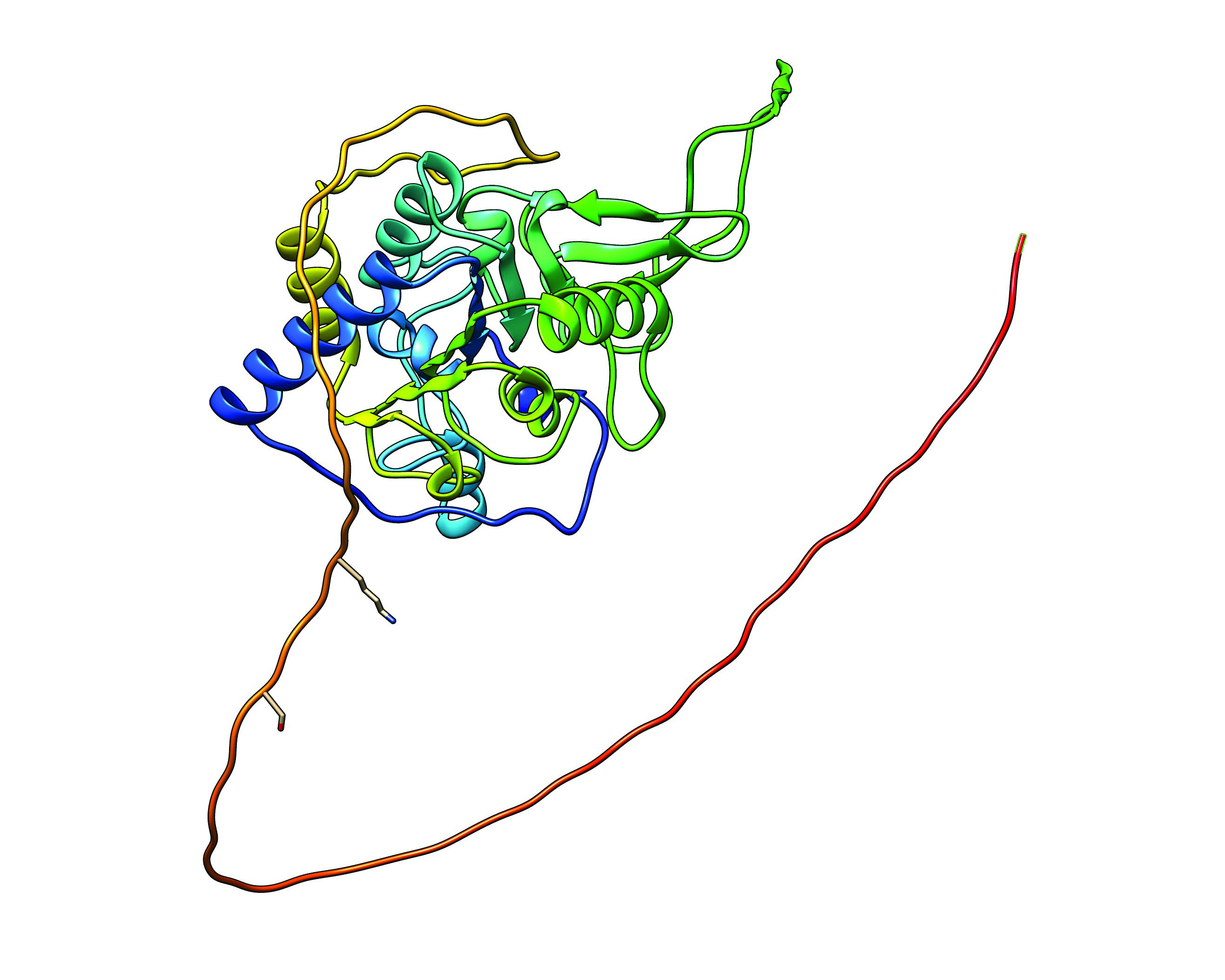


centSIRT6

N308K

A313S


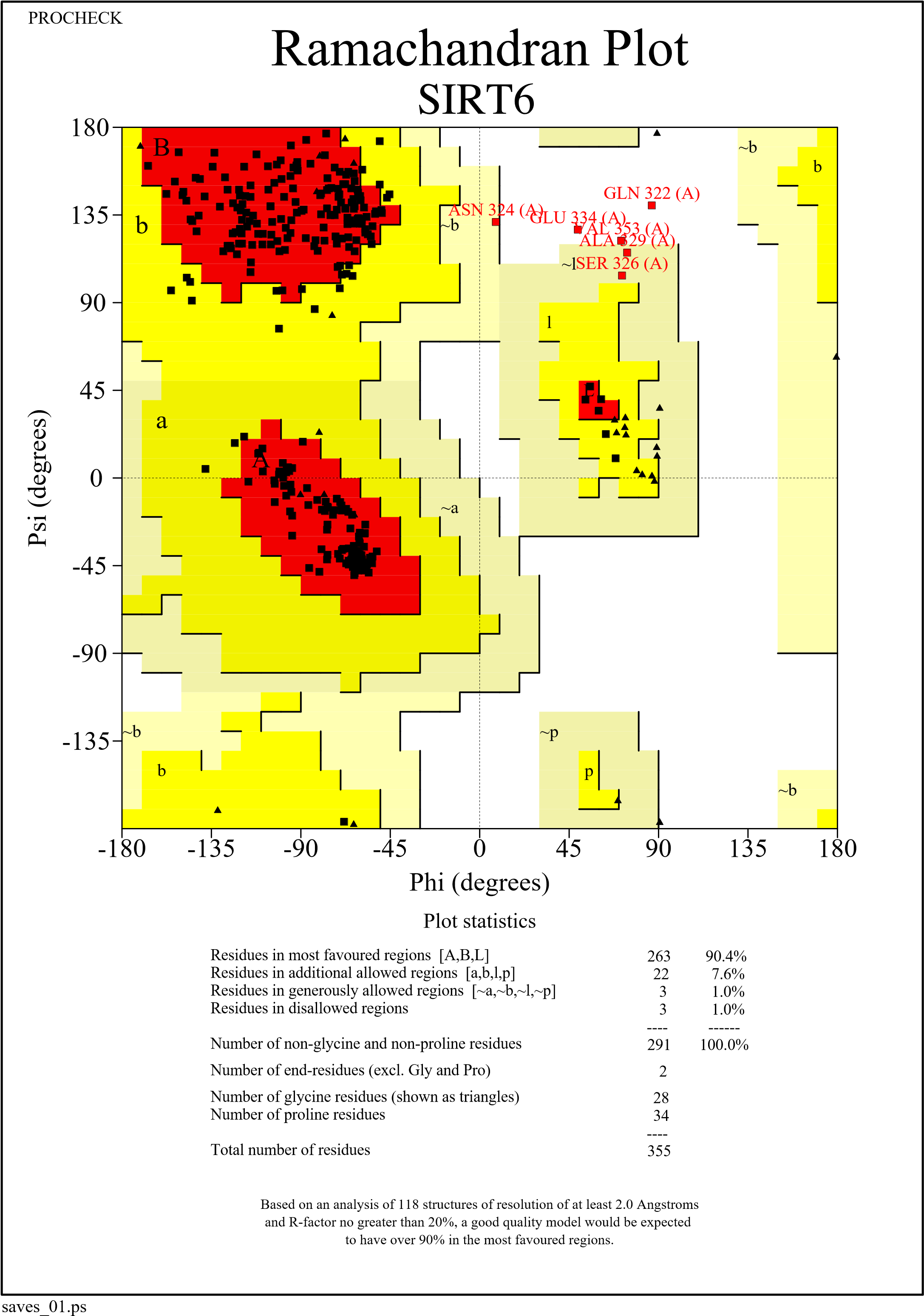


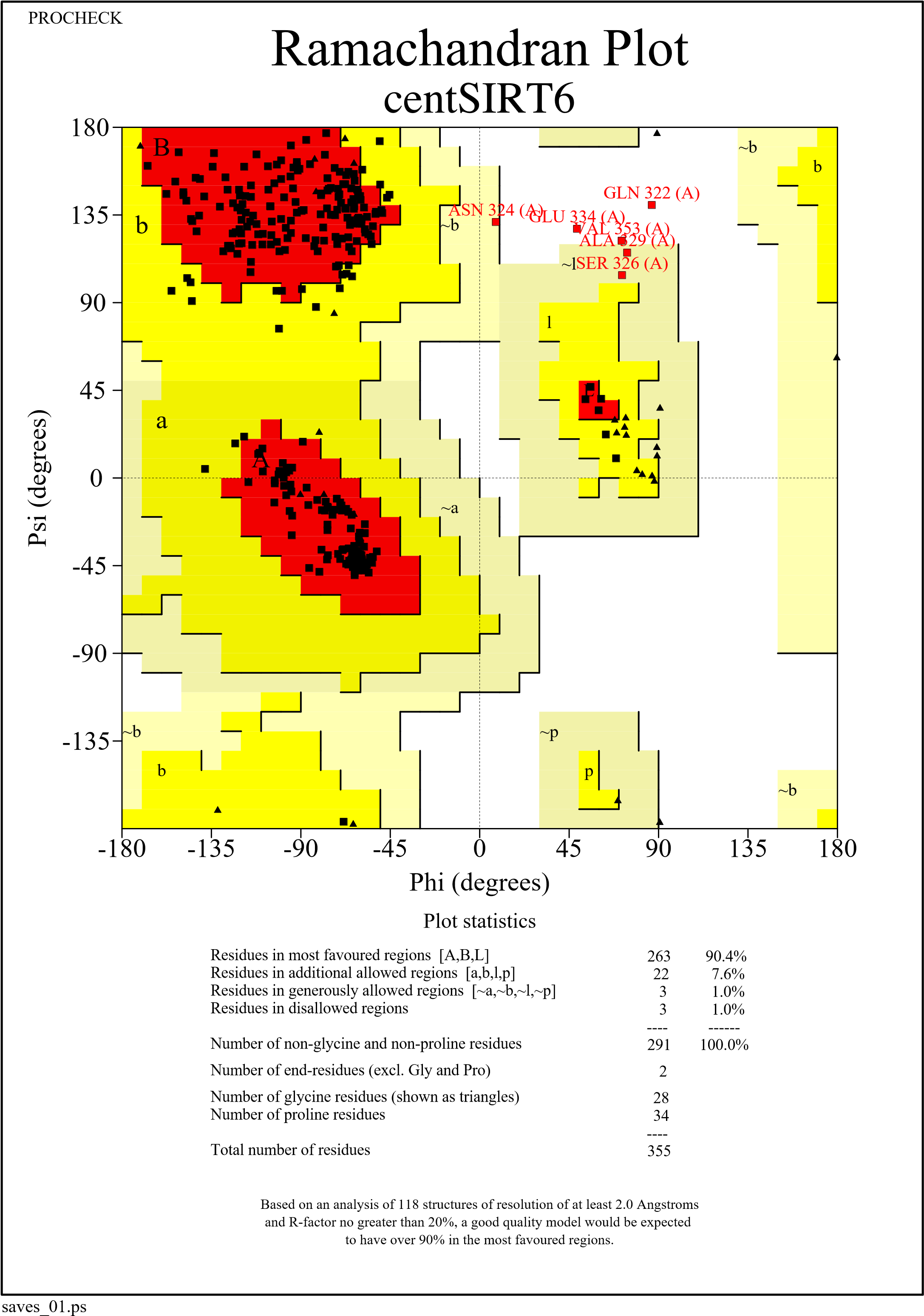

Supplement: sj-docx-1-bbi-10.1177_11779322251339698 – Supplemental material for Structural Insights Into centSIRT6: Bioinformatic Analysis of N308K and A313S Substitution Effects [file sj-docx-1-bbi-10.1177_11779322251339698.docx]
